# Visual Snow: Updates on Pathology

**DOI:** 10.1007/s11910-022-01182-x

**Published:** 2022-03-02

**Authors:** Clare L. Fraser

**Affiliations:** 1 Faculty of Health and Medicine, Save Sight Institute, The University of Sydney, 8 Macquarie Street, Sydney, NSW 2000 Australia; 2 Macquarie Ophthalmology, School of Clinical Medicine, Macquarie University, Sydney, Australia

**Keywords:** Visual snow, Palinopsia, Thalamocortical dysrhythmia, Inhibitory mechanisms, Cortical hyperexcitability, Migraine

## Abstract

**Purpose of Review:**

Until the last 5 years, there was very little in the literature about the phenomenon now known as visual snow syndrome. This review will examine the current thinking on the pathology of visual snow and how that thinking has evolved.

**Recent Findings:**

While migraine is a common comorbidity to visual snow syndrome, evidence points to these conditions being distinct clinical entities, with some overlapping pathophysiological processes. There is increasing structural and functional evidence that visual snow syndrome is due to a widespread cortical dysfunction. Cortical hyperexcitability coupled with changes in thalamocortical pathways and higher-level salience network controls have all shown differences in patients with visual snow syndrome compared to controls.

**Summary:**

Further work is needed to clarify the exact mechanisms of visual snow syndrome. Until that time, treatment options will remain limited. Clinicians having a clearer understanding of the basis for visual snow syndrome can appropriately discuss the diagnosis with their patients and steer them towards appropriate management options.

## Introduction

Patients with visual snow syndrome (VSS) report seeing continuous, uncountable, dynamic, tiny dots flickering across their entire visual field. They describe this as being like the static or the “snow” on a poorly tuned analog television. This “snow” is superimposed on the visual scene, and there is no loss of visual acuity nor a visual field defect [1, 2•]. Typically the static is black-and-white, but it can be coloured. While VSS could be thought of as a hallucination because there is no real-world correlate of the perception, it may be more accurate to consider it to be an illusion created by disordered visual processing. This hypothesis is supported by the presence of other visual phenomena that these patients often describe; persistence of afterimages, trail phenomena, sensitivity to bright light or night blindness; and an increased awareness of normal entoptic phenomena (visual perceptions whose source is within the eye itself, for example floaters) [3••, 4].

VSS is a disorder that is frequently overlooked or misdiagnosed. The lack of recognition of VSS, which was characterised only recently, has posted a challenge to clinicians and researchers. In our experience, for some patients, visual snow is extremely debilitating and results in behaviour like that seen with chronic disease. Some patients are unable to complete their schooling or participate actively in the workforce, and we have even seen patients placed on permanent disability pensions. Therefore, research and a better understanding of this condition will have an impact on patients’ quality of life and work.

### Diagnosis and Demographics

A set of diagnostic criteria for VSS has been proposed to capture the spectrum of the condition (Table 1) [5••]. The diagnosis of VSS can be made when a patient presents with subjective black-and-white visual static with at least one associated symptom of palinopsia (abnormal persistence or recurrence of an image in time), photopsia (flashes of light), nyctalopia (difficulty seeing in dim light or night time) and entoptic phenomena, excluding those that have a history more consistent with migraine aura or the symptoms occurring secondary to drug abuse. The population prevalence has been estimated to be 2% in the UK [6].

**Table 1:** Proposed criteria for visual snow syndrome [5••]

1	Visual snow: dynamic, continuous tiny dots in the entire visual field lasting longer than 3 months
2	Presence of at least two additional visual symptoms from the following: a) Palinopsia: afterimages or trailing of moving objects b) Photophobia c) Nyctalopia (impaired night vision) d) Other persistent positive visual phenomenon including (but not limited to) enhanced entoptic phenomena (excessive floaters or blue field entoptic phenomenon), kaleidoscope-type colours with eyes open or closed and spontaneous photopsias
3	Symptoms are not consistent with typical migraine visual aura
4	Symptoms are not better explained by another disorder

The average age of a large cohort of VSS patients was 29, though nearly 40% reported having symptoms for as long as they could remember. There was no sex prevalence found [2•]. However, papers have shown a female-to-male ratio of 1.6:1. [6], and in another 75% of VSS patients were male [7•]. On the severe end of the spectrum, patients experienced more types of associated visual symptoms [2•]. Some patients attributed symptom onset to a severe migraine attack. The prevalence of migraine and migraine with typical aura in VSS patients, 50–80%, is high in comparison with the general population, and migraineurs share some of the symptoms experienced by VSS patients, such as photopsias [2•]. Other patients report the onset of symptoms to be associated with certain medication, trauma or infection [2•]. A case of VSS presenting after SARS-CoV2 infection was published this year [8].

There have been reports of VSS from countries across the world. In one study comparing a population from the UK and Italy, the VSS phenotype and migraine comorbidity were similar [9]. Reports from Australia [7•], America [10], Israel [11] and Korea [12] are all remarkably similar.

### Visual Snow Mimics

Ophthalmic and other neurological conditions can present with features similar to VSS. While the absence of other disease processes is required for a diagnosis of VSS, the underlying pathophysiology of these conditions may be able to guide an understanding of what is, and what is not, VSS.

#### Ophthalmic Pathology

One explanation for the visual phenomenon of VSS is that they are ophthalmic in origin. Entoptic phenomena can also be experienced by those with otherwise healthy ophthalmic examinations because of vitreous floaters and white blood cells travelling through the microvasculature. Photopsia and other entopic phenomenon are reported in conditions such as posterior vitreous detachment, retinal tears, age-related macular degeneration (AMD) and retinitis pigmentosa [13]. Autoimmune retinopathy is characterised by circulating anti-retinal antibodies that cause photoreceptor dysfunction. Patients present with photophobia and positive visual scintillations that are often described as gold or silver shimmering in the visual periphery. The shimmering is most noticeable in the dark and persists with the eyes closed. A careful history can usually differentiate this from VSS, and diagnosis is based on fundus autofluorescence photography and electroretinography [14]. A case series of patients with glycine receptor autoimmunity describes symptoms of visual snow, palinopsia and positive visual phenomena which may be due dysregulation of the GlyRα1 inhibitory neurotransmitter in the human retina. [15]

In our own cohort of VSS patients, clinical examination and electrophysiology including electroretinography and visual evoked potentials were normal [7•]. In a Korean study of VSS patients, they also reported that findings are normal in patients with VSS, but on careful neuro-ophthalmic review, they found that one case had rod-cone dystrophy and another had idiopathic intracranial hypertension [12]. It is therefore unlikely that direct retinal pathology is a trigger for VSS, though the patients need thorough clinical review before a diagnosis is made.

#### Charles Bonnet Syndrome

Charles Bonnet syndrome (CBS) hallucinations can be characterised as simple flashes, dots of light or palinopsia. This phenomenon occurs in 40–60% of patients with profound bilateral loss of vision such as glaucoma or AMD [16]. The hallucinations of CBS are thought to arise from deafferentation of cortical areas which then become spontaneously active. Functional imaging suggests that the perceived images correspond to the functions of whichever cortical areas which are active at the time [17]. The different types of hallucination have been divided into sub-types corresponding to the anatomy of visual processing, for example delayed palinopsia (dorsal stream) [18]. More research is needed to see how these alterations in cortical activity in CBS can relate to VSS. The deafferentation of the visual system in CBS may be contributing to a form of thalamocortical dysrhythmia (see section below), in the same way as is described in tinnitus [19•].

#### Hallucinogen-Persisting Perception Disorder

Hallucinogen-persisting perception disorder (HPPD) represents spontaneous recurrence of visual perceptual disorders separated in time from the initial exposure to the hallucinogen. Recurrent hallucinations may take the form of geometric shapes, objects in the peripheral vision, flashes of different colours, trail phenomena, afterimages, stroboscopic perception of movement and/or disorders of size perception. Patients with HPPD tend to have a later onset of symptoms when compared to a cohort of VSS, and over 80% can pin point a specific onset of symptoms [2•]. In a recent review of HPPD, none of the patients reported migraine, compared to more than half of VSS controls [20].

Hallucinogen-persisting perception disorder has been reported in 5–50% of individuals exposed to LSD, sometimes lasting up to 5 years [21, 22]. Similar delayed visual symptoms have been reported following cannabis, often triggered by ethanol consumption or anaesthesia [23].

One possible cause of HPPD is excitotoxic damage of inhibitory interneurons. The LSD-generated currents may result in the destruction or dysfunction of cortical serotonergic inhibitory interneurons with gamma-aminobutyric acid (GABAergic) outputs. These are implicated in the sensory filtering mechanisms of unnecessary stimuli [24]. Given the symptomatic overlap, a deeper understanding of the pathophysiology of HPPD may help shed light on VSS.

## Migraine and Visual Snow

It was suggested in the mid-1990s that visual snow syndrome is a variant of migraine [25]. While many of the migraine features overlap with visual snow syndrome [26], visual phenomena are not directly linked to migrainous events, and the description of visual snow is clearly distinct from the typical content of migraine auras [25, 27]. There is, however, one series of 3 patients reporting episodic visual snow, co-occurring with migraine [28].

Migraine and migraine aura are reported in up to 80% patients with visual snow syndrome, higher rates than in the general population, which significantly complicates the phenotype [29••]. Furthermore, visual snow syndrome is more severe in individuals with a coexisting history of migraine [2•, 30]. The combination of migraine with VSS is associated with a three-fold increase in certain visual symptoms such as palinopsia and photopsias [3••].

Increased excitation of the serotonergic receptors has been documented in HPPD and migraine [3••, 31]. Although environmental triggers play a role, there is also strong evidence for a genetic predisposition to generalised neuronal hyperexcitability. The genes involved are regulators of synaptic transmission, glutamatergic excitation and plasticity for the development of cortical layers [31, 32]. This provides one possible mechanism to link migraine, HPPD and VSS.

A number of alterations in visual physiology and psychophysics have been reported in patients with migraine. Only about 10% of patients with migraine report palinopsia on direct questioning but formal testing for persistence of images after stimulus discontinuation suggest that this is present in over half of migraineurs, most frequently to the colour red [33]. Similarly, afterimage duration varies in migraineurs with their state: between migraine attacks, their afterimages disappear more quickly than in controls, but during a migraine attack, the afterimages last longer suggesting an alteration of excitatory status [34].

The movement of the aura across the visual field is consistent with spread of abnormal activity over the primary visual cortex at a rate of 2–3 mm/min [35], in-line with the cortical spreading depression hypothesis [36]. In patients reporting visual snow co-occurring with migraine, the snow was across the entire visual field and lacked a directed movement, which argued against the phenomenon being part of a cortical spreading depression-like mechanism [28].

Spread of increased blood-oxygen level-dependent (BOLD) activity followed by decreased activity has been demonstrated on fMRI with a time course corresponding to scintillations and scotoma [37], and similar changes have been demonstrated using SPECT, PET and MEG [38]. Patients with VSS show reduced BOLD responses to a visual stimulus on fMRI [39].

A reduced magnetic suppression of perceptual accuracy in the occipital cortex is seen in migraine patients with visual aura but appears not to be affected in visual snow [40].

A study using (18F)-FDG PET demonstrated that patients with VSS exhibits increase brain metabolism in the right lingual gyrus compared to healthy controls. The distribution of hypermetabolism was very similar to the area that shows to be linked to photophobia in migraine [41]. The authors concluded that this supports the pathophysiological overlap between the two conditions, at the same time as confirming that they are distinct syndromes.

## Potential Mechanisms of Visual Snow

The pathophysiology of visual snow is still under investigation. Given that VSS typically affects the entire visual field in both eyes, it is unlikely to be arising in the anterior visual pathways. Rather, it is likely to be a disorder of visual information processing. It has been hypothesised that similar pathophysiological mechanisms may account for both migraine and VSS and furthermore extend to other disorders of sensory processing such as tinnitus [42]. Achieving an improved understanding of the neurobiology of VSS will aid clinicians as they discuss the condition with their patients and will direct future research to targeted treatment options.

### Cortical Hyperexcitability

One theory to explain the neurobiological mechanism of VSS is that it is a purely cortical phenomenon. Visual disorders due to localised deficit or a region of hyperfunction in the V1/V2 areas of the occipital cortex can present with hallucinations with similarities to visual snow [43]. A lower threshold for occipital cortex excitability and a loss of habituation to transcranial magnetic stimulation phosphenes was seen in VSS patients compared to controls [44].

More recent studies have shown that patients with VSS have subtle, but widespread neuro-anatomical differences with an increase in grey matter volume in the left primary and secondary visual cortices and V5 visual motion area [45]. White matter abnormalities as detected with diffusion tensor imaging are also seen in dorsal and ventral streams in VSS patients [46]. On functional studies, patients also demonstrate higher regional cerebral blood flow over an extensive brain network when compared to controls [47]. In addition, fMRI of the resting-state functional connectivity found alterations in the brain regions involved in visual processing, memory, spatial attention and cognitive control in VSS patients [48]. These papers all suggest a more generalised neurobiological basis for VSS, rather than being purely occipital cortex involved.

Authors agree that there is a widespread dysfunction of higher-order visual processing areas, particularly the extrastriate cortex [29••]. One potential theory to explain the wider spread cortical mechanism suggests that stochastic resonance, a nonlinear phenomenon in which the addition of noise improves signal-to-noise ratio, improves the ability to detect a weak stimulus [49]. For example, it may be that coexisting tinnitus enhances the detection of visual stimuli in patients with visual snow syndrome, with one sensory system “priming” another [49]. Another possibility is that increased cortical neural excitability or visual pathway hypersensitivity leads in turn to perception of otherwise sub-threshold stimuli. In support of this, behavioural studies have demonstrated abnormal contrast and brightness processing in patients with visual snow syndrome [50].

Direct evidence for altered visual cortex excitability was shown by single case study using EEG [51]. Visual evoked potentials have shown prolonged latency and reduced amplitudes, which might suggest involvement of pre-striate pathways or the striate cortex itself [52]. PET scanning has demonstrated increased metabolism at the junction of the right lingual and fusiform gyrus [41], pointing to extrastriate cortical dysfunction. Other studies have shown that the lingual gyrus is involved in the perception of photophobia in migraine, consistent with the association between visual snow syndrome and migraine [53]. Using a combination of functional neuroimaging and magnetic resonance spectroscopy, differences were reported in the bilateral insular responses in VSS patients compared to controls, suggesting a localised disturbance in extrastriate anaerobic metabolism. It was hypothesised that this may in turn cause a decrease in the metabolic reserve for the regular processing visual stimuli [39]. Single-photon emission computed tomography (SPECT) in 3 VSS patients showed abnormal processing within the ventral visual stream in 2 of the patients [54].

Another area involved in the widespread dysfunction is likely to be the dorsal visual network or motion network which reaches from V1 dorsally to the parietal lobe and involves the motion area V5 in the temporo-parietal-occipital junction. Given the involvement in processing of visual motion, it is likely to play a role in the perception that the static dots are seen to be constantly moving. This region may also play a role in the trail phenomenon seen behind moving objects [55]. In the visually active state, the dorsal visual network and V5 showed hyper-integration to other brain areas in VSS patients [56••].

It is also hypothesised that VSS may result from general altered excitability and connectivity due to changes and altered connection in the brain networks involved in cognitive function. Heightened saccade-related activity in visual regions has been seen in VSS patients and may provide an objective clinical measure of this dysfunction [57]. In particular the salience network (SN) is thought to be responsible for detecting and filtering information necessary to maintain goal-directed behaviour. The SN increases activity in tasks requiring attention to external stimuli. This network refers to a group of brain regions located in the anterior cingulate and ventral anterior insular cortices and includes the thalamus [58]. The anterior insular cortex is critically involved in visual awareness [59] as well as emotional processing including anxiety [60]. Anxiety, depression and depersonalisation are frequent comorbid in a cohort of VSS patients [61].

A study of cortical functional connectivity has shown that VSS patients have a decreased connectivity during external sensory input within the salience network [56••]. This paper demonstrated widespread alteration in functional connectivity in VSS in both the resting and stimulated states. Regions within the visual network showed altered internal connectivity, as well as with basal ganglia and frontal eye fields. This dysfunctional salience may cause the brain to misattribute salience to internal stimuli that would otherwise be considered irrelevant causing the “noise-like” perception [56••].

### Thalamic Dysfunction

The thalamus is classically known for its role as a sensory relay in visual, auditory and somatosensory systems, as well as playing a role in consciousness and alertness. It is the lateral geniculate nucleus (LGN) that receives the visual sensory information from the retina to route to the visual cortex. The thalamic nuclei (excitatory and inhibitory) integrate these inputs and then present selected information to the cerebral cortex via thalamocortical radiations for interpretation [62].

The thalamus could be responsible for VSS symptoms through a localised increase in activity in the LGN or the pulvinar [29••]. The pulvinar has diffuse projections to the supragranular layers of the cortex and plays a role in attention and stimulus processing by aligning internal excitability patterns to the timing of relevant sensory inputs [63]. Reduced pulvinar connectivity to the visual cortico-striatal loop at rest has been found in VSS patients [56••]. Increased diffusivity on MRI has been reported in the thalamic radiations of VSS patients compared to controls [46]. During a visual task, heightened connectivity between the pulvinar and the lingual gyrus was reported which could explain a sensation of photophobia some patients describe, as well as causing a reduction in the filtering of incoming visual information [56••].

Oscillatory network activity is a characteristic property of that thalamocortical system and is central to cognitive processes such as attention and perception. An alteration of these oscillations, in particular an increase in the low-frequency delta and theta rhythms during states of wakefulness, is commonly termed thalamocortical dysrhythmia (TCD) [64]. When hyperexcitability affects cortical networks, as described in the section above, it can lead to TCD. Conversely, neuromodulatory processes involving the thalamus play a central role in how the brain modulates neural excitability [65]. This common underlying mechanism can produce a range of symptoms depending on the localization of the dysfunction in the thalamocortical network and may account for the spectrum of diseases associated with defaults in sensory processing [32]. Several apparently unrelated neurological conditions are thought to be a consequence of TCD, including migraine and tinnitus [66]. Thalamocortical dysrhythmia may therefore account for many of the comorbidities seen in visual snow syndrome such as tinnitus, impaired concentration, lethargy, anxiety, depression, tremor and balance disorders. All of these suggest that the underlying pathophysiology could represent a disorder of simultaneous processing of afferent information arriving at the cortex, not just in the visual domain [67]. Accordingly, the visual symptoms might simply represent a misperception rather than primary cortical hyperactivity [1].

Potentially, an underlying homeostatic imbalance of the visual pathways, from altered retinal activity, could cause a disinhibition of projections from the posterior thalamus to primary and secondary visual cortices [29••]. Imbalances between konio- and parvo/magnocellular pathway processing have previously been reported to underlie thalamocortical dysrhythmia in tinnitus and Parkinsonian tremor [68]. It is therefore hypothesised that koniocellular yellow-blue processing pathways [49] are also involved in VSS. The koniocellular pathway contains diffuse cortical connections via the LGN that modulate high-frequency cortical oscillations, thereby influencing sensory excitability [49]. The koniocellular pathways control slow cortical frequencies, in contrast to the parvo and magnocellular pathways which project to the primary visual cortex and are linked to fast cortical frequencies [69]. In support of this concept, wearing coloured visual filters helps some patients with visual snow syndrome, particularly those transmitting predominantly short (blue) wavelengths [7•]. Furthermore, VSS patients show a strong aversion to violet hues near the tritanopic confusion line, or S-cone axis, which increase S-cone excitation. Viewing a visual stimulus through this violet hue filter significantly exacerbated VSS symptoms. It is hypothesised that S-cone signals travelling in the koniocellular pathways contribute to dysregulation of the visual cortex via thalamo-cortical pathways [70].

Magnetoencephalography (MEG) is a non-invasive tool that is aimed at determining areas of metabolic activity and changes to cortical information spreading. MEG has been shown to identify and localise thalamocortical dysrhythmias in other disorders [68]. Alterations in the thalamic power-amplitude coupling to the visual cortex have been shown in visual snow patients compared to controls [71].

## Treatment Options

In general, VSS seems to be non-progressive, though it does fluctuate in severity within and between patients. Patients do not go blind, and they do not end up with a form of dementia. An explanation, reassurance regarding progress, acknowledging the data and the range of presentations to include sometimes significant disability, seems evidence-based and fair [72]. An honest discussion of the current state of research offers clarity and understanding which the patients generally find helpful. Some patients who are more prone to introspection and anxiety can struggle if they become too enmeshed in patient-led chat groups, so caution should be advised. However, others find the support of patients experiencing similar symptoms to be helpful.

In cases where the visual snow was brought on by an inciting event such as concussion, or infection, management of the underlying cause may significantly alleviate the otherwise intractable disturbances of VSS [10].

Studies report that some patients experienced partial improvement of VSS symptoms with drugs including benzodiazepines, lamotrigine, topiramate and acetazolamide [10]. Others report that of those listed, only lamotrigine afforded some improvement in a minority of patients [73]. However, on a large review of papers examining treatment in VSS, of the 44 medications tried, only 8 were effective at least once: lamotrigine, topiramate, valproate, propranolol, verapamil, baclofen, naproxen and sertraline. The best data was available for lamotrigine being effective in 8/36 (22%) cases, followed by topiramate being effective in 2/13 (15.4%). [74•]

Repetitive transcranial magnetic stimulation has shown conflicting results in patients with migraine [75]. However, it has not been widely tested in VSS. In one paper, a stimulus was applied to the visual cortex and compared to sham treatment in VVS patients. Treatment resulted in a trend to subjective improvement of visual snow intensity [76].

Tinted lenses have been showed to benefit patients suffering from migraine by reducing cortical hyperactivation [77]. Colorimetry assessment allows for adjustment of colour (hue) and depth of colour (saturation) with or without changes in associated luminance. The range of hues, saturation and luminance are tested, while a patient observes a visual target, such as a piece of text. Patients with VSS responded positively to coloured filters within the blue-yellow spectrum, reporting a subjective improvement in their symptoms [7•]. The use of tinted lenses has also been described elsewhere, though no data about the efficacy was presented [78].

To determine if treatments are providing any functional changes, rather than relying on subjective descriptions alone, it has been suggested that 3 simple ocular motor tasks could be used. Looking at prosaccade, antisaccade and interleaved pro-antisaccade tasks, an objective and quantifiable measure of the visual processing changes in VSS patients was recordable [79]. Other measures of treatment success also need to be determined before large-scale trials of any intervention are started.

Psychiatric symptoms are highly prevalent in patients with VSS and are associated with increased visual symptom severity and reduced quality of life [61]. Therefore treatment of psychiatric symptoms can offer an avenue for clinician to help the patients improve their quality of life and ability to cope with other symptoms. However, in a review of patients using serotonin reuptake inhibiting antidepressants, 8.9% reported visual snow, 10.5% palinopsia, 15.3% photophobia and 17.7% nyctalopia as a side effect of the medication [80]. Amitriptyline [74•] and citalopram [81] have also been reported to cause a worsening of VSS.

## Conclusion

**Fig. 1:**
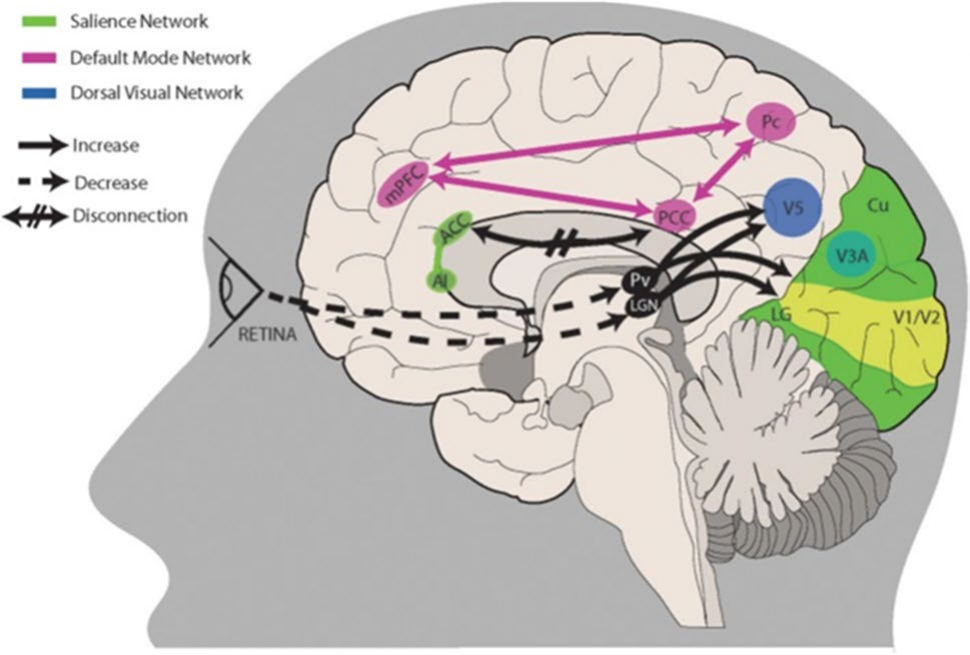
A proposed model for visual snow pathophysiology. Altered peripheral visual stimulation or a form of genetic predisposition could induce dysrhythmic connections between thalamic structures and cortical visual areas. The lateral geniculate nucleus (LGN) and pulvinar (Pv) in particular are directly connected to motion area V5 and the lingual gyrus (LG). Relevant to visual snow biology is the motion processing network, which is composed of areas within the primary visual cortex (V1/V2), area V3A within the cuneus (Cu), area V5 located ventrolaterally among the lateral occipital sulcus and inferior temporal sulcus, and Brodmann area 7 in the precuneus (Pc). Structures pertaining to the default mode network (PCC, posterior cingulate cortex; Pc; mPFC, middle prefrontal cortex) and/or the salience network (AI, anterior insula; ACC, anterior cingulate cortex) are involved in salience and interoception. Disruption of these networks, possibly through altered connectivity between cortical areas, could also play a role in visual snow pathophysiology. See main text for a more in-depth explanation. Available via open access – creative commons. https://www.ncbi.nlm.nih.gov/pmc/articles/PMC6923266/

It is most likely that the pathophysiology of visual snow syndrome is a combination of peripheral, thalamic and cortical dysfunction (Fig. 1) [29••]. The exact combination may vary slightly between patients, which could explain the main symptom of visual snow but the variety of other entoptic phenomena and indirectly related symptoms such as tinnitus. The generation of the persistent visual illusion could be a result of abnormal neurological activity in the thalamus and the visual system, otherwise normally ignored, and filtered from consciousness, being given increasing salience with no hierarchical network to then suppress the faulty perception. Further work is needed to clarify the interplay of these neurological systems and to begin to find targeted therapy to reduce the burden of this condition on VSS patients.
